# Characterization and Application of an Alginate Lyase, Aly1281 from Marine Bacterium *Pseudoalteromonas carrageenovora* ASY5

**DOI:** 10.3390/md18020095

**Published:** 2020-01-31

**Authors:** Yong-Hui Zhang, Yuan Shao, Chao Jiao, Qiu-Ming Yang, Hui-Fen Weng, An-Feng Xiao

**Affiliations:** 1 College of Food and Biological Engineering, Jimei University, Xiamen 361021, China; yhz@jmu.edu.cn (Y.-H.Z.); 201711710036@jmu.edu.cn (Y.S.); 201711710029@jmu.edu.cn (C.J.); yangqm@jmu.edu.cn (Q.-M.Y.); wwhhffen2020@163.com (H.-F.W.); 2 Fujian Provincial Engineering Technology Research Center of Marine Functional Food, Xiamen 361021, China; 3 Xiamen Key Laboratory of Marine Functional Food, Xiamen 361021, China

**Keywords:** alginate lyase, brown seaweed, antioxidant, salt activation, molecular dynamics

## Abstract

Alginate extracted from widely cultured brown seaweed can be hydrolyzed by alginate lyase to produce alginate oligosaccharides (AOS) with intriguing biological activities. Herein, a novel alginate lyase Aly1281 was cloned from marine bacterium *Pseudoalteromonas* *carrageenovora* ASY5 isolated from mangrove soil and found to belong to polysaccharide lyase family 7. Aly1281 exhibited maximum activity at pH 8.0 and 50 °C and have broad substrate specificity for polyguluronate and polymannuronate. Compared with other alginate lyases, Aly1281 exhibited high degradation specificity and mainly produced di-alginate oligosaccharides which displayed good antioxidant function to reduce ferric and scavenge radicals such as hydroxyl, ABTS^+^ and DPPH. Moreover, the catalytic activity and kinetic performance of Aly1281 were highly improved with the addition of salt, demonstrating a salt-activation property. A putative conformational structural feature of Aly1281 was found by MD simulation analysis for understanding the salt-activation effect.

## 1. Introduction

Alginate is a linear water-soluble polysaccharide that accounts for up to 40% of the dry weight of brown seaweed [1]. Alginate polysaccharide is composed of (1,4)-linked-d-mannuronic acid (M) and *R*-l-guluronic acid (G) with different structural arrangements (e.g., homopolymeric, MM–, GG–, or heteropolymeric, MG–, GM–). Due to the widespread cultivation of brown seaweed in Asia, alginate has been extensively studied for its extraction ability and is widely used as a viscosifier, stabilizer, and gelling agent in the food [2], chemical [3] and biomaterials [4] industries. Many recent studies have demonstrated that alginate oligosaccharides (AOS), which exhibit outstanding biological properties that differ from those of alginate polysaccharides, can be produced by further hydrolysis of alginate; in particular, AOS presents anti-antioxidant [5], anticoagulant [6] and antineoplastic [7] properties. Some studies have shown that AOS can lower blood sugar and blood lipid levels [8] and display anti-tumor [9] and anti-viral [10] abilities. Moreover, they can be used as prebiotic to promote the growth of probiotics, such as *Bifidobacterium bifidum*, *Bifidobacterium longum* [11], and *Lactobacilli* [12].

Alginate lyase can catalyze the degradation of alginate polysaccharides through a β-elimination reaction to cleave glycosidic bonds and produce AOS [13]. Thus, the development for alginate lyase is crucial to efficiently generate AOS. For example, AOS prepared by the lyase of *Streptomyces* sp. strain A5 shows growth-promoting activity in the roots of banana plantlets [14]. Zhu et al. [15] effectively produced oligosaccharides with 2–5 degrees of polymerization (DP) using a new endo-type alginate lyase from *Vibrio* sp. W13. Alginate lyases are mainly isolated from marine-derived organisms, including animals, bacteria, algae, fungi, and viruses [16,17]. Marine-derived alginate lyases often exhibit considerable salt-tolerance ability or salt-activation effects [3,9,15,18] due to the high-salt marine environment in which the source bacteria are reproduced and evolve. The alginate lyase derived from marine *Vibrio* Harveyi-28, for example, exhibits a 24-fold increase in activity in 1 M NaCl [19]. Despite the extensive development of alginate lyases and their widely observed salt-regulated properties, investigations into the salt-activation mechanism of alginate lyase are scarce.

In the present work, the novel alginate lyase Aly1281 was cloned from *Pseudoalteromonas carrageenovora* ASY5 and expressed in *Escherichia coli*. Its enzymatic properties were then characterized. Aly1281 can be used to hydrolyze sodium alginate, producing mainly di-alginate oligosaccharides, and exhibits high degradation specificity compared with other alginate lyases. In addition, the antioxidant capacity of the obtained AOS was investigated. A comparative molecular dynamics (MD) study was performed to study the salt-activation effect of Aly1281, as the lyase is significantly activated by addition of salt ions (Na^+^, K^+^).

## 2. Results and Discussion

### 2.1. Aly1281 Gene and Protein Sequence Information

**Figure 1:**
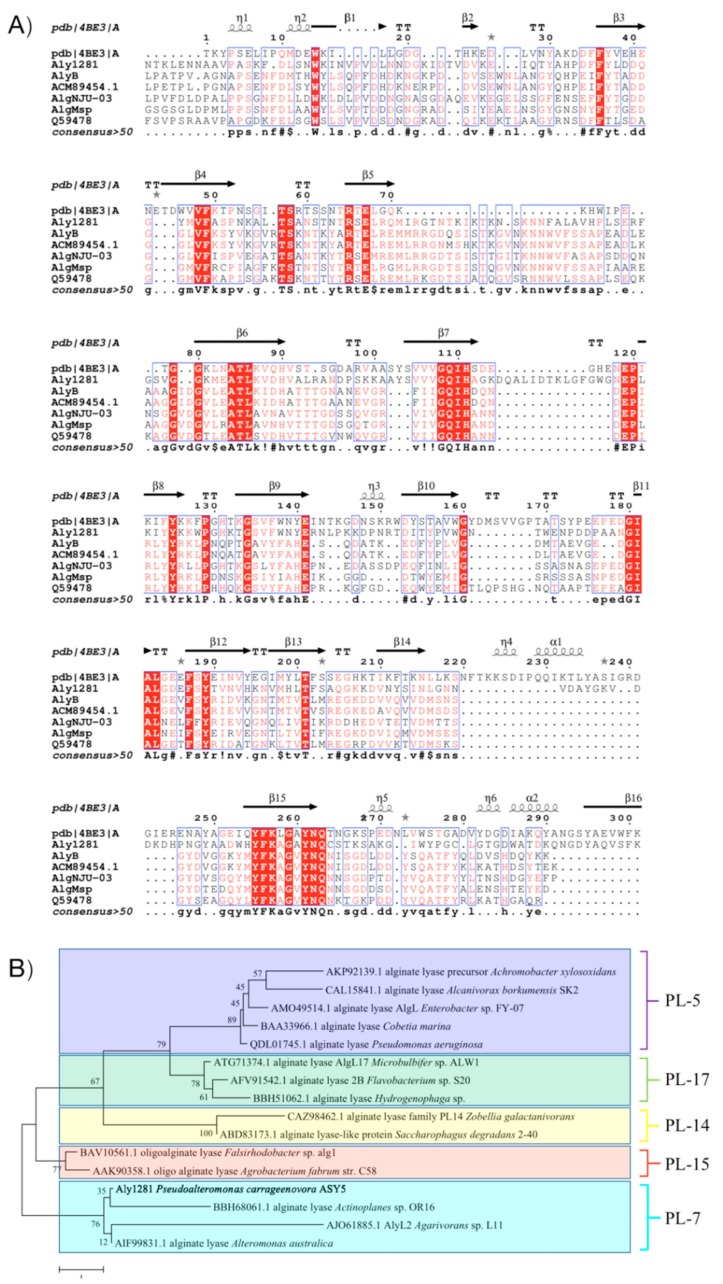
Multiple sequence alignment and phylogenetic analysis of A1y1281. (**A**) Conserved regions are highlighted with a yellow border. Residues involved in the catalytic and formation of substrate-binding pockets are indicated by green and blue triangles, respectively. (**B**) A phylogenetic tree was constructed by using MEGA software version 7.0 (MEGA7). Alginate lyases from families 5, 7, 14, 15, and 17 are respectively marked by purple, blue, yellow, red, and green boxes.

The alginate lyase gene was cloned, sequenced, and found to consist of 1089 bp. Its open reading frame (ORF) encoded 361 amino acids with a signal peptide of 26 amino acids predicted by using SignalP (http://www.cbs.dtu.dk/services/SignalP). The mature enzyme had a calculated molecular mass of 40.04 kDa and a predicted pI value of 9.06. The sequence alignments of Aly1281 and the previous characterized alginate lyases of PL7 are shown in Figure 1A. The alignment indicated that Aly1281 was most closely related to alginate Lyase (PDB ID: 4BE3_A) from *Zobellia galactanivorans* with an amino acid sequence identity of 42.4%. Aly1281 contained three highly conserved regions of PL family 7, namely, R(S/T)ELV(R/G), QIH, and YFK(A/L)GAYNQ, which played an important role in the substrate binding and catalytic activity of the enzymes [15,17,20]. Moreover, catalytic (Q173, H175, and Y315) and substrate binding (R104, E194, K197, E216, D228, and W327) sites were predicted in Aly1281 on the basis of previous research on alginate lyase (AlyA5) from *Z. galactanivorans* [21]. A phylogenetic tree was constructed (Figure 1B) according to the different families of alginate lyases, and the result showed that Aly1281 is located in the same clade with PL7 alginate lyases including BBH68061.1, AJO61885.1, and AIF99831.1. In summary, the results of sequence alignment and phylogenetic analysis imply that Aly1281 belongs to PL family 7.

### 2.2. Expression, Purification and Biochemical Characterization of Recombinant Enzyme Aly1281

**Figure 2:**
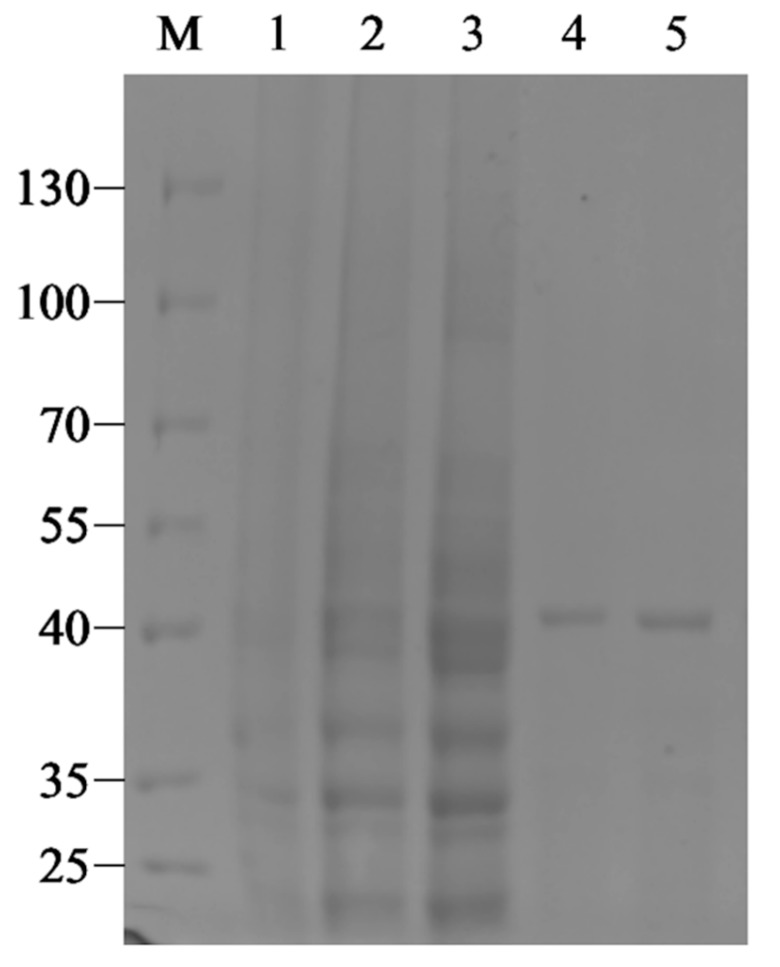
SDS-PAGE profiles of crude and purified Aly1281. M lane, protein marker; Lane 1, blank control expressing empty pET-28a plasmids; Lanes 2–3, crude Aly1281; Lane 4–5, purified Aly1281.

The recombinant strain BL21(DE3)/pET28a-Aly1281 was successfully induced by IPTG to produce recombinant Aly1281, which was purified by a Ni-NTA agarose column. The recombinant enzyme yield was 2.5 mg per liter of growth media. The theoretical molecular mass of the expressed Aly1281 was 40.65 kDa based on the nucleic acid sequence of the enzyme. The result of SDS-PAGE (Figure 2) was consistent with the predicted theoretical molecular mass. The enzymatic activity of Aly1281 was 1.15 U/mg. The purified Aly1281 was treated with thrombin to cut the His-tag and the result indicated that the enzyme activity of Aly1281 was not affected by His-tag. Therefore, the purified Aly1281 was used in further investigations of enzymatic characteristics.

**Figure 3:**
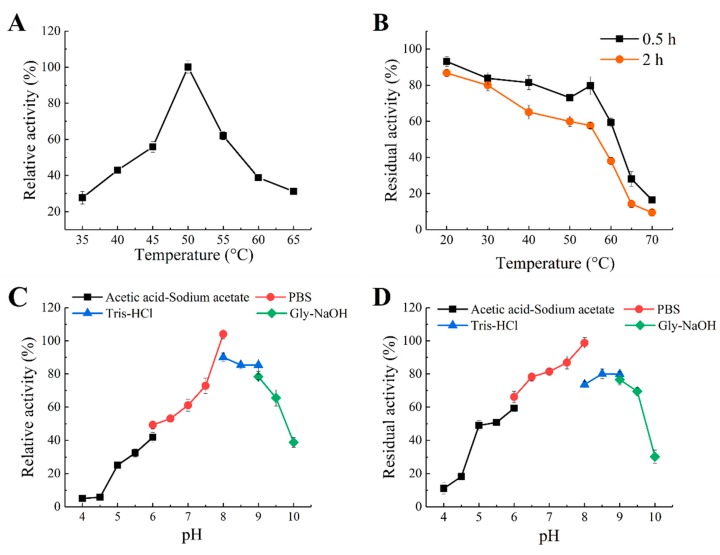
Effects of pH and temperature on the activity and stability of Aly1281. (**A**) Optimal temperature: Aly1281 activities were determined at various temperatures (35–65 °C). (**B**) Thermal stability: The residual activities of Aly1281 were determined after incubation at various temperatures (0–70 °C) for 30 min or 2 h. Relative activity is expressed as a percentage of maximum activity under the experimental conditions. (**C**) pH optima: Aly1281 activities were determined in the acetic acid-sodium acetate buffer (pH 4.0–6.0), Na_2_HPO_4_–NaH_2_PO_4_ buffer (pH 6.0–8.0), Tris–HCl buffer (8.0–9.0), and Gly–NaOH buffer (pH 9.0–10.0). (**D**) pH stability: The residual activities of Aly1281 were measured after the enzyme was incubated in the pH range of 4.0 to 10.0 with the above buffers for 24 h at 4 °C. Data represent the mean ± standard deviation of triplicate measurements.

The effect of temperature on the activity of Aly1281 was shown in Figure 3A. Aly1281 exhibited maximum enzyme activity at 50 °C. At the temperature range of 45–55 °C, Aly1281 manifested over 50% of the activity observed at the optimal temperature. Few studies had reported alginate lyases that exhibited optimal reaction temperatures higher than 50 °C, such as AMOR_PL7A (65 °C) [22], cAlyM (55 °C) [23] and Alg823 (55 °C) [5]. The thermostability of Aly1281 was determined at a temperature ranging from 0 °C to 70 °C. As shown in Figure 3B, Aly1281 was relatively stable at temperatures lower than 55 °C. Aly1281 showed competitive thermostability in comparison with most reported marine-derived alginate lyases [15,24,25,26], which could benefit its industrial application. Zhu et al. [3] characterized an extracellular biofunctional alginate lyase that retained less than 50% of the initial activity after incubation at 45 °C for 2 h. Li et al. [24] purified a new alginate lyase that retained less than 30% of the initial activities after incubation at 45 °C for 2 h.

The optimum reaction pH of Aly1281 was determined by assaying enzyme activities using buffers of different pH (4.0–10.0). As shown in Figure 3C, the optimum reaction pH of Aly1281 is 8.0. Aly1281 exhibited over 65% relative activity in solutions of neutral and weakly alkaline pH (6.0–9.5). Aly1281 activity was nearly negligible at pH below 4.5, which indicates its sensitivity to acidic reaction conditions. These results are similar to those obtained from most reported marine-derived alginate lyases, which exhibit optimal pH at 6.0–9.0 [24,26,27]. The pH stability of Aly1281 was evaluated by measuring the residual activity after storing Aly1281 in buffers with different pH (4.0–10.0) for 24 h at 4 °C (Figure 3D). The Aly1281 was most stable at pH 8.0. Aly1281 could retain more than 70% and 50% of the relative residual activities at pH ranges of 7.0 to 9.0 and 5.0 to 9.5, respectively.

### 2.3. Substrate Specificity and Degradation Pattern of Aly1281

The substrate specificity of Aly1281 was studied by using three substrates. Aly1281 possessed catalytic abilities toward all tested substrates, including alginate (100%), polyG (134.32%), and polyM (53.88%). Moreover, Aly1281 exhibited higher activity toward polyG than toward polyM. Similar results have been reported for alginate lyase from *Isoptericola halotolerans* CGMCC5336 [9] and *Microbulbifer* sp. 6532A [18]. Some reported alginate lyases are only specific for polyM or polyG [6,14]. The results indicate that Aly1281 has broad substrate specificity and is a bifunctional alginate lyase that hydrolyzes both polyG and polyM.

**Figure 4:**
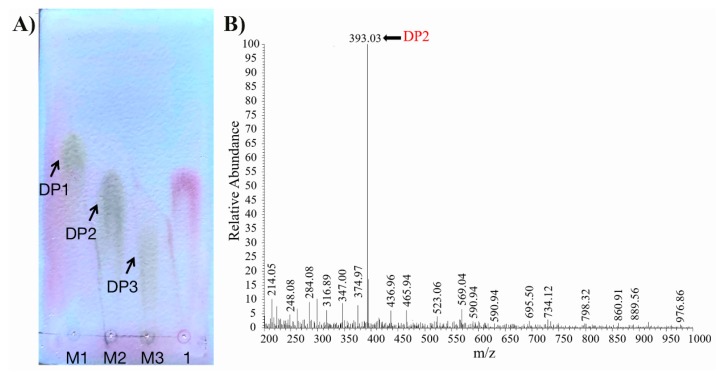
(**A**) TLC analysis of the hydrolysis products of Aly1281 with alginate sodium. Lanes 1–3 refer to the DP1–3 standards. (**B**) ESI-MS analysis of the hydrolysis products of Aly1281with alginate sodium.

After 12 h of hydrolysis, the enzymatic degradation products of Aly1281 were analyzed by using TLC and ESI-MS. As shown in Figure 4A,B, the main product of the sodium alginate hydrolysis reaction is di-alginate oligosaccharide, which indicates that Aly1281 is an endo-type alginate lyase. Compared with other alginate lyases from different sources which produced alginate oligosaccharides with broad DP, the degradation products of Aly1281 displayed highly specific degradation [15,19]. Aly510-64 from *Vibrio* sp. 510-64, which produces only tri-alginate oligosaccharides, exhibited a similar level of degradation specificity [28]. The high degradation specificity of Aly1281 is particularly appropriate for the preparation of AOS with high purity and could be beneficial for the production of high-value-added alginate products.

### 2.4. Antioxidant Function of the Degradation Products of Aly1281

AOS has attracted wide attention due to its unique properties, such as anti-aggregatory [29], antioxidant [30], antitumor [31] effects, and ability to regulate blood sugar and lipids [15]. AOS reduces osteosarcoma progression and may be used as a potential drug for osteosarcoma therapy [31]. Tusi et al. [32] confirmed the neuroprotective potential of AOS against Ab-induced neural damage. Moreover, AOS has been demonstrated to render the heart resistant to I/R injury by inhibiting nitrative/oxidative stress and ER stress-mediated apoptosis [33]. These reports collectively suggest a connection between oxidative stress and apoptosis. Thus, we investigated the antioxidant effect of AOS by using various approaches, which include ferric reducing, hydroxyl radical scavenging, ABTS radical scavenging, and DPPH radical scavenging assays.

**Figure 5:**
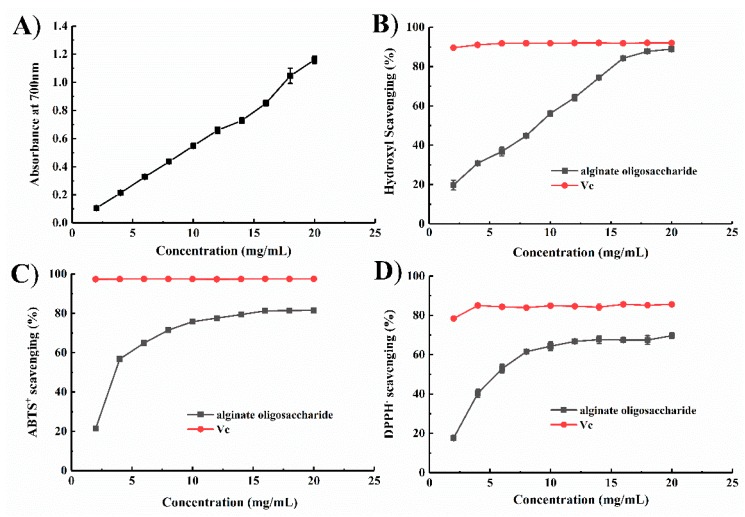
Antioxidant function of alginate oligosaccharides. (**A**) Reducing ability. (**B**) Scavenging effect on hydroxyl radicals. (**C**) Scavenging effect on ABTS radicals. (**D**) Scavenging effect on DPPH radicals. Data represent the mean ± standard deviation of triplicate measurements.

A direct correlation between antioxidant activities and reducing power has been reported [34]. Fe^3+^ reduction ability is an important index of electron-donating activity [35]. As shown in Figure 5A, the reducing effects of AOS increased from 0.105 to 1.16 at 700 nm with increasing enzyme concentration. The reducing ability of AOS is generally associated with the presence of reductones, which have been shown to exert antioxidant action by breaking free radical chains by donating a hydrogen atom [36]. The results indicate the ability of AOS to reduce ferric ions to ferrous ions.

Hydroxyl radicals were produced in vivo from water by high-energy irradiation or from H_2_O_2_ via a metal-catalyzed process which led to lipid peroxidation, massive protein oxidation and degradation, and DNA damage [37,38,39,40]. Most of the harmful effects of these radicals can be significantly reduced by addition of hydroxyl radical scavengers. In the present work, the hydroxyl radical scavenging activity of AOS was determined by using salicylic acid as the molecular probe. AOS exhibited a concentration-dependent ability to scavenge hydroxyl radicals and showed a maximum activity of 88.87% ± 0.31% when applied at a concentration of 20.0 mg/mL (Figure 5B). Moreover, the EC50 of the hydrolysates was 8.7 mg/mL, which is lower than those of other alginate oligosaccharides reported by Zhu et al. [3].

DPPH and ABTS radicals are also used as substrates to evaluate the free radical scavenging ability of an antioxidant [41]. The groups involved in H-atom transfer reactions toward DPPH and ABTS^+^ radicals are mainly C-2 and C-6 hydroxyls [42]. In the present work, the oligosaccharide samples were able to scavenge ABTS^+^ and DPPH free radicals even at low concentrations. As shown in Figure 5C,D, 20 mg/mL AOS exhibited optimal ABTS^+^ and DPPH radical scavenging activities of 81.50% ± 0.33% and 69.65% ± 1.40%, respectively. The EC50 values of AOS for ABTS^+^ and DPPH radicals were 5.65 and 7.84 mg/mL, respectively, which are higher than those of the oligosaccharides reported in [43]. Chien et al. [44] showed that, compared with medium- or high-molecular-weight chitosan oligosaccharides, low-molecular-weight chitosan oligosaccharides exhibited higher free radical scavenging activity. The hydrolysis by Aly1281 resulted in mainly low-molecular-weight AOS (di-alginate oligosaccharide), which ensured high free radical scavenging activity.

Falkeborg et al. [40] purposed that radical scavenging activity of AOS mainly originates from a conjugated alkene acid structure. In addition, Xu et al. [45] purposed the catalytic mechanism of alginate lyase for the formation of the alkene acid structure based on structural and mutational analysis. In summary, the AOS obtained from Aly1281 presents good scavenging activities of up to >88%, >81%, and >69% toward hydroxyl, ABTS, and DPPH radicals, respectively. AOS production by the enzymatic hydrolysis of Aly1281 had great potential in the high-value-added processing of marine seaweed resources.

### 2.5. Salt Activation of Aly1281

**Figure 6:**
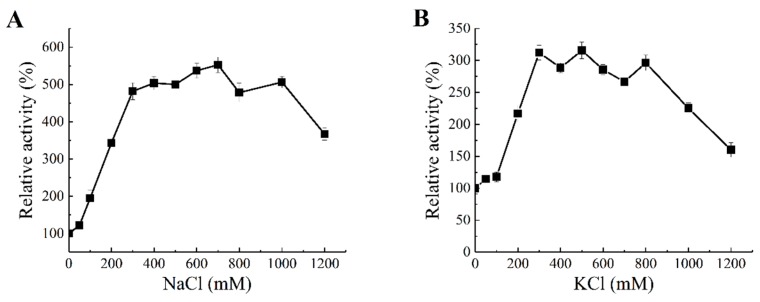
Effects of (**A**) NaCl and (**B**) KCl concentration on the enzymatic activity of Aly1281. Aly1281 activities were measured by using standard conditions as described in the Methods section. The activity observed without salt addition was considered to be 100% for calculations of relative activities. Each value represents the mean of three replicates ± standard deviation. Data represent the mean±standard deviation of triplicate measurements.

Due to the unique properties of the marine environment where the sourced bacteria are reproduced and evolve, enzymes extracted from marine bacteria often exhibit interesting enzymatic properties, such as salt tolerance and salt activation. The concentration effects of salts including NaCl and KCl on the enzyme activity of Aly1281 are shown in Figure 6. The enzyme activity of Aly1281 peaked after addition of 700 mM NaCl and 300 mM KCl, which, respectively, increased the activity of the lyase by 4.56- and 2.77-fold compared with that without salt addition. Aly1281 activity increased sharply with addition of NaCl and KCl at concentrations lower than 300 mM. Addition of excess NaCl and KCl at concentrations higher than 300 mM showed limited influence on enzyme activity. Moreover, addition of over 1000 mM salt reduced enzyme activity levels to a greater extent compared with that obtained after addition of salt at low concentrations. These results indicate that the proper amount of salt could greatly promote Aly1281 activity. The catalytic efficiency of Aly1281 was highly activated by salt concentrations similar to the average salt concentration in the ocean (ca. 430 mM). Thus, Aly1281 could be applied in the industrial enzymatic processing of brown seaweed without water-intensive desalting [46].

The effect of salt addition (300 mM NaCl and 1000 mM KCl) on the kinetic parameters of Aly1281 was evaluated by using sodium alginate as a substrate (Table 1). Addition of 300 and 1000 mM NaCl decreased *K*_m_ by 54.9% and 74.3%, respectively, compared with the values observed without salt addition. Similarly, addition of 300 and 1000 mM KCl decreased the *K*_m_ value by 60.3% and 76.9%, respectively, relative to the no-salt case. Moreover, the *k*_cat_/*K*_m_ of Aly1281 substantially increased by salt addition. When 300 and 1000 mM NaCl were added to the system, the *k*_cat_/*K*_m_ of Aly1281 increased by 353% and 664%, respectively, relative to the control. When 300 and 1000 mM KCl were added, the *k*_cat_/*K*_m_ of Aly1281 increased by 341% and 508%, respectively, relative to the control. The observed kinetic performance of Aly1281 indicates that salt addition could greatly enhance the substrate affinity and catalytic efficiency of the lyase, which may explain the salt activation effect.

Alginate lyases of difference sources exhibit different levels of salt-activation effects and optimal salt concentrations. For example, the activity of AlgNJU-03 from *Vibrio sp.* NJU-03 is not affected by Na^+^ or K^+^ [47], the activity of Algb from *Vibrio sp.* W13 is increased by 2.2-fold by 0.3 M NaCl [15], and the activity of alginate lyase from *Microbulbifer sp.* ALW1 is increased by 5.1-fold by 0.5 M NaCl [3]. Indeed, the activity of alginate lyase from *Vibrio.* Harveyi-28 enhanced by 24-fold by 1 M NaCl [19]. In the present work, the activity of Aly1281 from *P. carrageenovora* ASY5 increased by 5.56-fold after addition of 0.7 M NaCl. According to previous halophilic mechanism studies [48,49,50], the divergence of salt-activation effects among alginate lyases could be related to the bacterial source, protein sequence, and structure.

### 2.6. Structural Insight into the Salt-Regulated Conformational Dynamic of Aly1281

**Figure 7:**
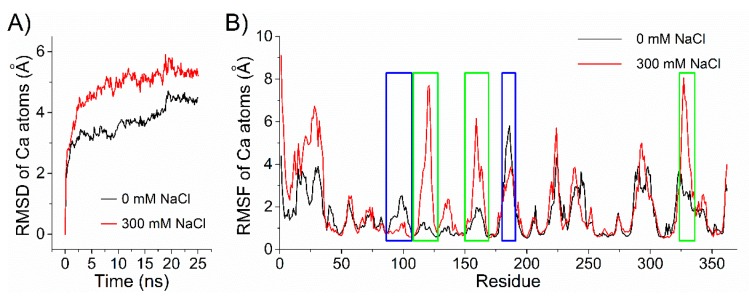
(**A**) RMSDs of alpha carbon atoms in 25 ns MD simulations as a function of time relative to the initial structure of Aly1281. (**B**) RMSFs of the alpha carbon atoms in 25 ns MD simulations of Aly1281. Blue and green boxes indicate protein domains that are highly stabilized or flexibilized by the addition of salt.

Enzyme catalysis has been proven to be highly correlated with its conformation dynamics [51,52,53]. The salt-activated or -dependent properties of alginate lyase may be due to its salt-regulated conformational dynamics. A preliminary investigation of the salt activation effect of Aly1281 was conducted by using MD simulations. RMSDs in 25 ns MD simulations were calculated by using the initial structure of the lyase as a reference (Figure 7A). Aly1281 achieved stable states with average RMSDs of ~4.4 Å without NaCl and ~5.3 Å in the presence of 300 mM NaCl. Aly1281 showed higher RMSDs in 300 mM NaCl than in the absence of NaCl over the entire MD simulation process. This result indicates that addition of salt increases the overall structural fluctuations of Aly1281. Similar increases in RMSD in *Candida antarctica* lipase B in high-concentration urea solvent have been observed [54]. The RMSFs of the MD simulations are shown in Figure 7B; here, different protein domains of Aly1281 responded differently to the addition of salt. The domain conformations could be either stabilized or flexibilized by salt addition.

**Figure 8:**
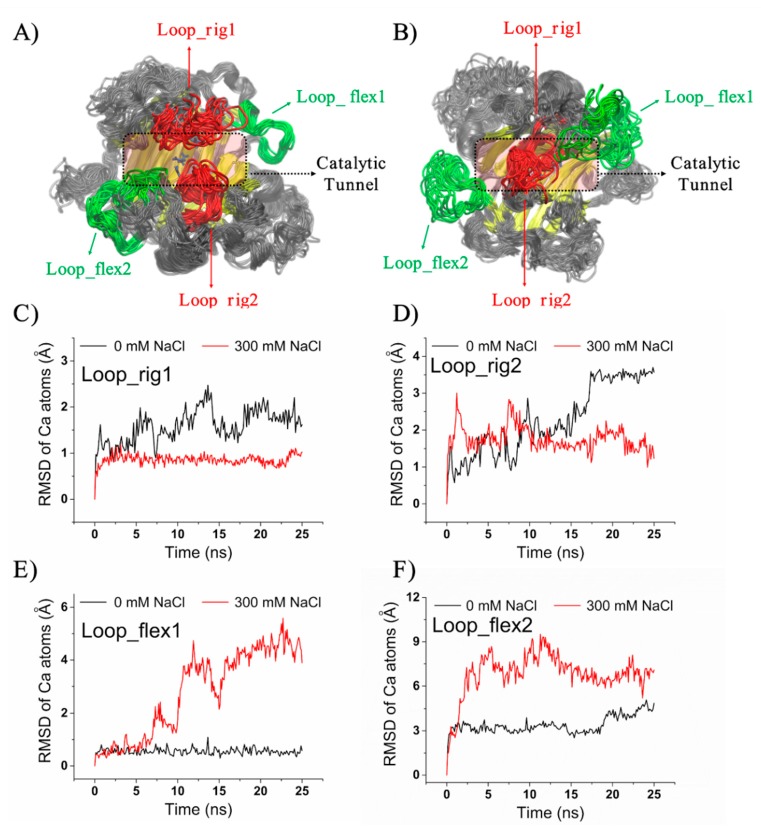
Conformational dynamics of Aly1281 in 25 sequential frames extracted from 25 ns MD trajectories with (**A**) 0 and (**B**) 300 mM NaCl. Catalytic tunnel-forming β-sheets are shown in yellow. The catalytic residues Q173, H175, and Y315 are shown as blue sticks. Two highly stabilized regions, namely, Loop_Rig1 and Loop_Rig2, are depicted in red, and two highly flexibilized regions, namely, Loop_Flex1 and Loop_Flex2, are displayed in green. RMSDs of the alpha carbon atoms of (**C**) Loop_Rig1, (**D**) Loop_Rig2, (**E**) Loop_Flex1, and (**F**) Loop_Flex2 in 0 and 300 mM NaCl refer to the initial energy-minimized structures.

MD trajectories for Aly1281 were characterized in terms of RMSFs from the energy-minimized structure to further study potential salt-induced conformational changes. Figure 8A,B show the conformational dynamic patterns of Aly1281 in the presence of 0 and 300 mM NaCl. The domains most influenced by the addition of salt showed significantly enhanced or depressed fluctuations and mainly comprised Loop_Rig1 (_90_SPNKALTSANSTNTRSE_106_) and Loop_Rig2 (_181_ALIDTKLG_188_), which were particularly stabilized by the addition of salt, and Loop_Flex1 (_113_GTNTKIKTKNSKNN_126_) and Loop_Flex2 (_156_LRANDPSKKAA_166_ and _326_IWYPG_330_), which were flexibilized by the salt solvent environment. According to a previous study on the crystal structure and catalytic mechanism of alginate lyase [21], Loop_Rig1 (_90_SPNKALTSANSTNTRSE_106_) and Loop_Rig2 (_181_ALIDTKLG_188_) are lid loops located at opposite sides of the catalytic tunnel and near substrate binding sites (i.e., Q173, H175, and Y315). These lid loops are a typical structural feature of PL7 and PL18 alginate lyases and play critical roles in their catalytic reaction. A diversity study of alginate lyases, including AlyA from *K.* *pneumoniae* (PDB code 4OZX), alyPG from *Corynebacterium* sp. ALY-1 (PDB code 1UAI), and AlyA1 (PDB code 3ZPY) and AlyA5 (PDB code 4BE3) from *Z. galactanivorans*, demonstrated the possible relationship between the lid loop architecture (open or close) and cleavage functional variations (endolytic or exolytic activity) [55]. The RMSDs of Loop_Rig1 and Loop_Rig2 during the simulations were calculated using the initial structure as reference (Figure 8C,D). Loop_Rig1 achieved stable states with average RMSDs of ~1.7 Å without NaCl and ~0.8 Å in the presence of 300 mM NaCl. Loop_Rig2 achieved stable states with average RMSDs of ~3.5 Å without NaCl and ~1.4 Å in the presence of 300 mM NaCl. These results indicate that the conformational fluctuations of these loops are stabilized by the addition of salt. Moreover, unlike the open conformation of Loop_Rig1 and Loop_Rig2 with 0 mM NaCl, Loop_Rig1 and Loop_Rig2 appear to show a closed conformation near catalytic residues in the presence of 300 mM NaCl. Considering the MD simulation results, the salt-induced conformational dynamics of lid loops may be involved in the salt-activation of Aly1281.

Loop_Flex1 and Loop_Flex2 of Aly1281, which are located near the gateway of the catalytic tunnel, showed increased conformational fluctuations in the presence of 300 mM NaCl (Figure 8E, F). Loop_Flex1 achieved stable states with average RMSDs of ~0.5 Å without NaCl and ~4.4 Å in the presence of 300 mM NaCl. Loop_Flex2 achieved stable states with average RMSDs of ~4.6 Å without NaCl and ~7.0 Å in the presence of 300 mM NaCl. These results indicate that the conformational fluctuations of these loops are rendered flexible by the addition of salt, especially in Loop_Flex1, which showed an eightfold enhancement of RMSD. The salt-regulated flexibilization of these “entrance” loops may facilitate product release and lead to increases in catalytic efficiency. Improvements in catalytic efficiency by tunnel variation have also been observed in the rational engineering of epoxide hydrolase, where two amino acid substitutions could expand the product-release tunnel and enhance activities by 42- and 25-fold compared with that of the wild-type enzyme [56]. Despite the significant increase in fluctuation of these loops; however, the gyration radius of Aly1281 is only slightly increased from ~20.8 Å (without salt) to ~21.3 Å (with 300 mM NaCl) (Figure S1), which reflects a stable structural integrity in salt environments. In summary, the salt-activation effect of Aly1281 revealed a putative conformational dynamic feature. Future experimental validation and broader analysis of other similar enzymes will be performed to obtain an accurate understanding of the salt-activation mechanism of alginate lyase from the PL7 family.

## 3. Materials and methods

### 3.1. Strains and Materials

*P. carrageenovora* ASY5 (CCTCC No. CICC23819) was isolated from a mangrove (Xiamen, China) and stored in the College of Food and Biological Engineering of Jimei University. pET-28a(+) vectors, *Escherichia coli* DH5α and BL21 (DE3) strains were purchased from TAKARA (Beijing, China). DNA polymerase, protein molecular weight markers, and polyacrylamide were purchased from TransGen Biotech (Beijing, China). Sodium alginate (Lot no. S26286), d-polymannuronic acid (polyM, Lot no. B25951) and l-polyguluronic acid (polyG, Lot no. B25962) was purchased from Yuanye Bio-Technology (Shanghai, China). Other chemicals used in this study were of analytical grade.

### 3.2. Construction of Recombinant E. coli

The Aly1281 gene from the genomic DNA of *P.* *carrageenovora* ASY5 was used to design primers for PCR amplification. The forward primer was 5ʹ-CCGGAATTCAATACTAAACTGGAAAA-3ʹ, and the reverse primer was 5ʹ-CCCAAGCTTCGGCTTAGTGCTCGGGC-3ʹ. The Aly gene was obtained via PCR amplification using the genomic DNA of *P. carrageenovora* ASY5 as a template. The PCR products were analyzed by agarose gel electrophoresis and then purified and digested with BamHI and EcoR I. The recovered Aly gene was ligated with T4 DNA ligase to pET-28a (+) plasmids digested with the same restriction enzymes and then transformed into *E. coli* BL21 (DE3) competent cells for enzyme expression.

### 3.3. Expression and Purification of Aly1281

The constructed recombinant *E. coli* containing the Aly1281 gene was inoculated in LB medium containing 50 mg/L kanamycin at 37 °C with horizontal shaking at 180 rpm. When the OD_600_ of the solution reached 0.6–0.8, 0.5 mmol/L IPTG was added to induce Aly1281 expression. The cells were allowed to grow at 16 °C with horizontal shaking at 180 rpm for 20 h, collected via centrifugation, sonicated in lysis buffer (300 mM NaCl, 15 mM imidazole, 50 mM NaH_2_PO_4_), and then centrifuged at 8000 rpm for 20 min at 4 °C to obtain crude Aly1281. The crude enzyme was loaded onto a Ni-NTA agarose column equilibrated with lysis buffer. The column was washed with washing buffer (300 mM NaCl, 15 mM imidazole, 50 mM NaH_2_PO_4_) to remove protein impurities, and the recombinant Aly1281 was eluted with elution buffer (300 mM NaCl, 250 mM imidazole, 50 mM NaH_2_PO_4_). Eluted fractions with Aly activity were collected, combined and the buffer was replaced with Tris–HCl buffer (50 mM, pH 8.0) using a Macrosep Advance centrifugal device (Pall, East Hills, NY, USA; cut-off distance, 10 kDa), and then analyzed by 12% sodium dodecyl sulfate polyacrylamide gel electrophoresis (SDS-PAGE).

### 3.4. Measurement of A1y1281 Activity

Exactly 200 μL of purified recombinant enzyme Aly1281 was mixed with 800 μL of sodium alginate (0.5%, pH 8.0) and allowed to react at 50 °C for 40 min. The reaction was terminated by using a boiling water bath. Then, 1 mL of 3, 5-dinitrosalicylate (DNS) solution was added to the solution, and the mixture was allowed to react in a boiling water bath for 10 min. Absorbance was measured at 540 nm. One unit of Aly1281 activity was defined as the amount of enzyme required to catalyze the production of 1 μmol of reducing sugar per minute.

### 3.5. Effects of Temperature and pH on Aly1281 Activity and Stability

The activities of alginate lyase at different temperatures (35–65 °C) were measured to investigate the effect of temperature on Aly1281 activity; relative enzyme activities were calculated by using the activity obtained at the optimum temperature as 100%. Aly1281 was incubated at different temperatures (30–70 °C) for 2 h to investigate the effect of temperature on Aly1281 stability; here, residual enzyme activities were measured under the assay conditions described previously and calculated by using the initial activity as 100%.

Alginate lyase activities in different buffers, including acetic acid–sodium acetate buffer (50 mM, pH 4.0–6.0), Na_2_HPO_4_–NaH_2_PO_4_ buffer (50 mM, pH 6.0–8.0), Tris–HCl buffer (50 mM, pH 8.0–9.0), and glycine–sodium hydroxide buffer (50 mM, pH 9.0–10.0), were measured under the assay conditions described previously to investigate the effect of pH on Aly1281 activity; here, relative enzyme activities were calculated by using the activity obtained at the optimum pH as 100%. Aly1281 was incubated in the buffers described above (50 mM, pH 4.0–10.0) at 4 °C for 24 h to investigate the effect of pH on Aly1281 stability; here, residual enzyme activities were measured under the assay conditions described previously and calculated by using the highest residual activity as 100%.

### 3.6. Effects of Salts on the Activity and Kinetic Parameters of Aly1281

The effects of salts on Aly1281 activity were measured under the assay conditions described previously with different concentrations of NaCl or KCl (0–1200 mM); relative enzyme activities were calculated by using the activity obtained without salts as 100%. The kinetic parameters of the purified Aly1281 at various salt concentrations were studied by assaying the enzyme activity of sodium alginate in different salt concentrations (0.5–5 mg/mL). The Lineweaver–Burk method was applied to calculate *K*_m_ and *k*_cat_.

### 3.7. Substrate Specificity of Aly1281

The substrate specificity of purified Aly1281 was investigated by measuring its activity under the assay conditions described previously with 2 mg/mL sodium alginate, d-polymannuronic acid (polyM) and l-polyguluronic acid (polyG) as substrates.

### 3.8. TLC and ESI-MS Analysis of the Degradation Products of Aly1281

Exactly 200 µL of purified enzyme was added to 800 µL of the substrate solution (5 mg/mL sodium alginate) to enable the degradation reaction of Aly1281. The degradation reaction was conducted at 50 °C for 12 h, and the reducing sugar content in the reaction mixture was determined periodically. When a stable reducing sugar content was achieved, the reaction mixture was boiled for 10 min and centrifuged at 12,000 rpm for 10 min. Absolute ethanol was then added to the supernatant to a final concentration of 75% (v/v), and the mixture was incubated at 4 °C for 2 h. After incubation, the supernatant was concentrated by rotary evaporation and lyophilized.

The degradation products of Aly 1281 were analyzed by using TLC with a solvent of *n*-butyl alcohol–acetic acid–water (2:2:1). Visualization of the products was achieved by spraying the TLC plate with 10% (v/v) H_2_SO_4_ in alcohol and then heating at 120 °C for 5 min.

The composition and DP of the products were further determined via electrospray ionization mass spectrometry (ESI-MS). A total of 2 µL of the degradation products was loop-injected into the ESI-MS instrument (Bruker Esquire HCT, USA) operated in negative-ion mode with the following settings: calibration dynamics, 2; capillary voltage, 4.00 kV; cone voltage, 20.00 V; source temperature, 150 °C; desolvation temperature, 350 °C; cone gas flow rate, 50 L/h; and desolvation gas flow, 500 L/h.

### 3.9. Antioxidant Function of the Alginate Degradation Products of Aly1281

#### 3.9.1. Ferric Reducing Power

Ferric reducing power was determined in accordance with a previously described method [57] with slight modifications. Briefly, 300 μL of degradation products of different concentrations was mixed with 350 μL of 0.2 M sodium phosphate buffer (pH 7.0) and 350 μL (1%, w/v) of [K_3_Fe(CN)_6_]. After 20 min of incubation at 50 °C, the mixture was added to a mixture of 350 μL (10%, w/v) of trichloroacetic acid and 150 μL (1%, w/v) of FeCl_3_ (0.1%). The absorbance of the mixture was measured at 700 nm by using a UV spectrophotometer. Distilled water was used as the blank.

#### 3.9.2. Scavenging Activity of Hydroxyl Radical

Hydroxyl radical scavenging activity was determined in accordance with a previously described method [5]. Briefly, 0.1 mL of FeSO_4_ (9.0 mM), 0.6 mL of deionized water, 2 mL of oligosaccharide solution, 0.1 mL of H_2_O_2_ (8.8 mM), and 0.1 mL of ethanol salicylate (9.0 mM) were mixed and incubated at 37 °C for 30 min. The absorbance of the mixture was then determined at 510 nm. Distilled water and Vc were used as the blank and positive control, respectively. Scavenging activity (%) was calculated by using the following equation:

Hydroxyl free-radical scavenging activity (%) = (A_0_ − A_sample_)/A_0_ × l00, (1)

where A_0_ and A_sample_ are the absorbance of the blank and final absorbance of each sample at 510 nm, respectively.

#### 3.9.3. Scavenging Activity of 2,2-Diphenyl-1-picrylhydrazyl (DPPH)

DPPH radical-scavenging activity was determined by using a previous method with minor modifications [58]. Oligosaccharide solutions (500 μL) of different concentrations were mixed with DPPH reagent (500 μL) and then incubated in the dark at room temperature for 30 min. The absorbance of the mixture was determined at 517 nm. Distilled water was set as the blank, and Vc was used as the positive control. The scavenging activity (%) of DPPH was calculated by using the following equation:

DPPH radical scavenging activity (%) = (A_0_ − A_sample_)/A_0_ × l00, (2)

where A_0_ and A_sample_ are the absorbance of the blank and final absorbance of each sample at 517 nm, respectively.

#### 3.9.4. Scavenging Activity of 2,2′-Azinobis-(3-ethylbenzthiazoline-6-sulphonate) (ABTS)

ABTS radical-scavenging activity was determined according to a previous method with minor modifications [59]. ABTS (7 mM) and K_2_S_2_O_8_ (2.45 mM) solutions were mixed at equal volumes and stored in the dark for 16 h to produce ABTS radicals (ABTS^+^). The mixture was diluted with PBS (pH 7.0) to an absorbance of A_734nm_=0.700 ± 0.020. Then, 0.1 mL of oligosaccharide solution and 1 mL of ABTS solution were mixed and incubated at 37 °C for 15 min. The absorbance of this solution was measured at 734 nm. Distilled water was set as the blank, and Vc was used as the positive control. The scavenging activity (%) of ABTS was calculated by using the following equation:

ABTS radical scavenging activity (%) = (A_0_ − A_sample_)/A_0_ × l00, (3)

where A_0_ and A_sample_ are the absorbance of the blank and final absorbance of each sample at 734 nm, respectively.

### 3.10. Comparative Study by MD Simulation

Modeling of the 3D structure of Aly1281 was conducted by using the web-based I-TASSER server (http://zhanglab.ccmb.med.umich.edu/I-TASSER) using AlyB from *Vibrio splendidus* (PDB ID: 5zu5), alginate lyase from *Klebsiella pneumoniae* (PDB ID: 4ozx), and AlyA5 from *Zobellia galactanivorans* (PDB ID: 4be3) as threading templates [60]. MD simulations were performed to investigate the salt-induced conformational dynamics of Aly1281 and conducted by using the NAMD 2.13 simulation software package. The CHARMM36 force field was used. The TIP3P water model was used for solvent molecular modeling. Na^+^ and Cl^−^ were used to neutralize charges in the simulation system and added to the required concentration for NaCl simulation. The particle mesh Ewald algorithm was used to calculate long-range electrostatic interactions with cutoff distances of 12 Å for non-bonding interactions and 10 Å for switching distances. The MD simulations were performed under NPT conditions using Langevin dynamics and the Langevin piston method to maintain a constant pressure and temperature. Hydrogen atoms between proteins and water molecules were kept rigid by using the RATTLE and SETTLE algorithms. Simulations were carried out at a 2 fs timestep, and coordinates were saved at 10 ps intervals. Each simulation system was first minimized to relax high-energy contact with 15,000 energy minimization steps at 50 K followed by 25 ns simulations in the presence of 0 or 0.3 M NaCl at 323.15 K. Simulation trajectories were generated, and physical parameters, including root-mean-squared deviation (RMSD), root-mean-square fluctuation (RMSF) and radius of gyration were calculated by using VMD. Protein structure images were obtained by VMD.

## 4. Conclusions

In the present research, we describe the characterization of the novel PL7 alginate lyase Aly1281 from the marine bacterium *P. carrageenovora* ASY5. Aly1281 showed maximum activity at 50 °C and pH 8.0. The enzyme has broad substrate specificity and could endolytically cleave alginate. Aly1281 exhibited high degradation specificity toward sodium alginate and mainly produced di-alginate oligosaccharides. The degradation products of Aly1281 exhibited good antioxidant activity, thus indicating the potential applications of this lyase in the food industry. A putative conformational structural feature was proposed by MD simulation analysis to better understand the salt-activation effect of Aly1281. Future work will focus on the determination of the 3D structure of the lyase under different salt concentrations to enable the broader analysis of other similar enzymes and accurate elucidation of the salt-activation mechanism of Aly1281.

## Figures and Tables

**Table 1:** Kinetic parameters of Aly1281 at different NaCl or KCl concentrations.

Salt Addition	Km/(mg/mL)	kcat/(s^−1^)	kcat/Km/(s^−1^·mg^−1^·mL)
Control	0.7065	1.070	1.515
0.3 M NaCl	0.3180	2.185	6.871
1.0 M NaCl	0.1810	2.095	11.576
0.3 M KCl	0.2805	1.875	6.685
1.0 M KCl	0.1631	1.502	9.208

## Supplementary Materials

The following are available online at https://www.mdpi.com/1660-3397/18/2/95/s1, Figure S1: The radius of gyration of Aly1281 during 25 ns MD simulation as a function of time.
