# Supplementary material for: Characterization and Application of an Alginate Lyase, Aly1281 from Marine Bacterium Pseudoalteromonas carrageenovora ASY5
Source: Mar Drugs. 2020 Jan 31;18(2):95. doi: 10.3390/md18020095 (PMC7073683; doi:10.3390/md18020095)
Supplement: Supplementary file 1 [file marinedrugs-18-00095-s001.pdf]

Supplementary Material

Characterization and application of an alginate lyase, Aly1281 from Marine bacterium *Pseudoalteromonas carrageenovora* ASY5

Yong-Hui Zhang[a,b,c #], Yuan Shao[a,b,c #], Chao Jiao[a,b,c], Qiu-Ming Yang[a,b,c], Hui-Fen Weng[a,b,c] and An-Feng Xiao[a,b,c *]

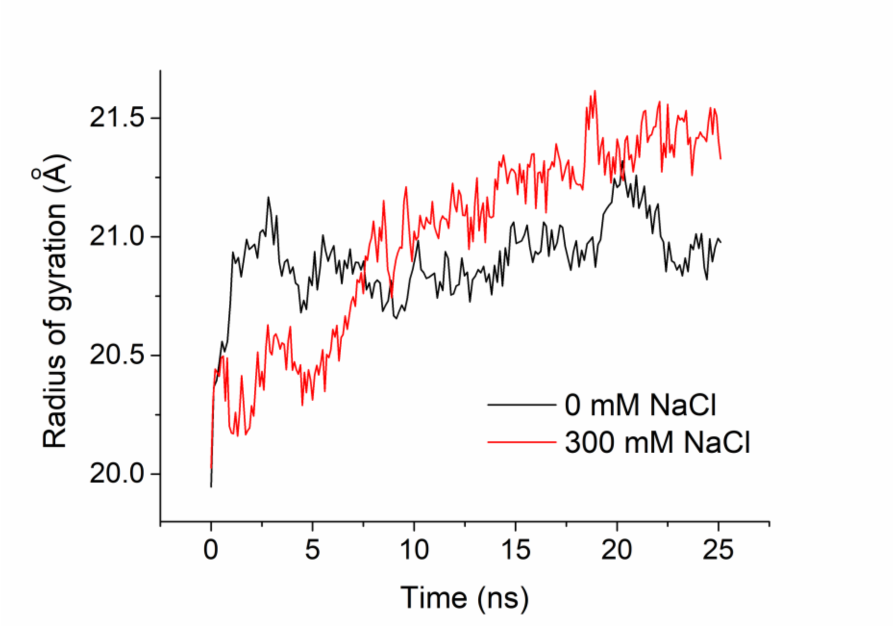

Figure S1. The radius of gyration of Aly1281 during 25 ns MD simulation as a function of time.
